# Developing machine-learning-based models to diminish the severity of injuries sustained by pedestrians in road traffic incidents

**DOI:** 10.1016/j.heliyon.2023.e21371

**Published:** 2023-10-31

**Authors:** Amir Elalouf, Slava Birfir, Tova Rosenbloom

**Affiliations:** a Bar-Ilan University, Department of Management, Ramat-Gan 52900, Israel; b Elbit Systems Company, Haifa 3100401, Israel

**Keywords:** Pedestrian injury severity, Classification machine-learning algorithms, Feature list, Road safety, Prediction

## Abstract

An essential step in devising measures to improve road safety is road accident prediction. In particular, it is important to identify the risk factors that increase the likelihood of severe injuries in the event of an accident. There are two distinct ways of analyzing data in order to produce predictions: machine learning and statistical methods. This study explores the severity of road traffic injuries sustained by pedestrians through the use of machine-learning methodology.

In general, the goal of the statistician is to model and understand the connections between variables, whereas machine learning focuses on more intricate and expansive datasets, with the aim of creating algorithms that can recognize patterns and make predictions without being explicitly programmed. The ability to handle very large datasets constitutes a distinct advantage of machine learning over statistical techniques. In addition, machine-learning models can be adapted to a wide range of data sources and problem domains, and can be utilized for numerous tasks, from image identification to natural language processing. Machine-learning models may be taught to recognize patterns and make predictions automatically, minimizing the need for manual involvement and enabling rapid data processing of enormous quantities of data. The use of new data to retrain or fine-tune a machine-learning model allows the model to adapt to changing conditions and enhances its accuracy over time. Finally, while non-linear interactions between variables can be difficult to predict using conventional statistical techniques, they can be recognized by machine-learning models.

The study begins by compiling an inventory of features linked to both the accident and the environment, focusing on those that exert the greatest influence on the severity of pedestrian injuries. The “optimal” algorithm is then chosen based on its superior levels of accuracy, precision, recall, and F1 score. The developed model should not be regarded as fixed; it should be updated and retrained on a regular basis using new traffic accident data that mirror the evolving interplay between the road environment, driver characteristics, and pedestrian conduct. Having been constructed using Israeli data, the current model is predictive of injury outcomes within Israel. For broader applicability, the model should undergo retraining and reassessment using traffic accident data from the pertinent country or region.

## Highlights

• Compiling a list of features for diverse machine-learning algorithms aimed at predicting the classification of pedestrian injury severity.
• Identifying the optimal machine-learning algorithm.
• Pedestrian crash severity prediction.

## 1 Introduction

### 1.1 The scope of the investigation

**Fig. 1:**
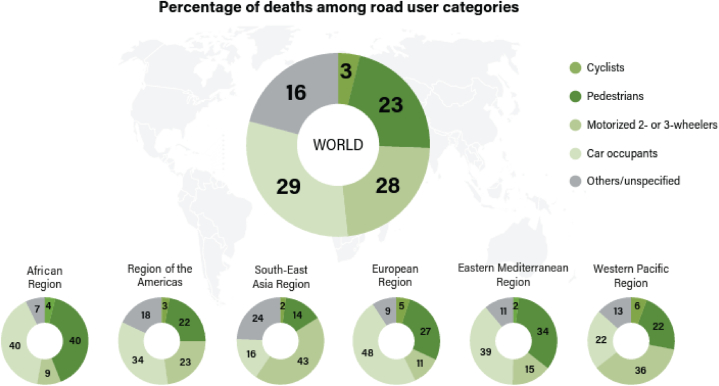
Proportion of fatalities among different road user categories, extracted from the road safety manual for decision-makers and practitioners [59].

Pedestrians are widely acknowledged as one of the most vulnerable subgroups of road users. According to a pedestrian safety report [59], road safety for pedestrians remains a concern on a global scale. Worldwide, the rate of pedestrian fatalities has escalated almost twice as fast as overall road crash fatalities, with a 12.9 % increase from 2013 to 2016, as opposed to 6.6 % for other road users. Approximately 310,500 pedestrians lost their lives in road traffic accidents across the globe in 2016, constituting around 23 % of all road traffic fatalities (see Fig. 1). Within the United States of America, the period between 2008 and 2018 witnessed a 41 % rise in pedestrian deaths, while fatalities for other road users experienced a reduction of 7 %. According to the US National Highway Traffic Safety Administration [4], in 2019, an average of one pedestrian was fatally injured in traffic crashes every 88 min. Further reinforcing this concern, two recent reports by the Governors Highway Safety Association [2,3] highlighted a 53 % surge in pedestrian fatalities in the US in 2018 compared with 2009.

Notably, Fig. 1 shows that pedestrian fatalities exhibit a distinct geographical distribution, with the proportion of pedestrian deaths (out of all road-user fatalities) being highest in the African Region (40 %) and lowest in the South-East Asian Region (14 %). In India, pedestrian fatalities account for approximately 30 % of the total deaths resulting from road traffic incidents [14]. The proportion of pedestrian fatalities in major cities, such as New Delhi and Mumbai, ranges from 50 % to 60 % of all road traffic fatalities, while on national and state highways, it stands at 20 %–30 % [14]. A recent examination encompassing nine cities in India revealed that the percentage of road traffic fatalities involving pedestrians spanned from 22 % to 46 % of the overall death count [59].

This study endeavors to develop a classification model using machine learning aimed at forecasting the severity of pedestrian injuries stemming from traffic accidents. The model is based on three severity categories – fatal, serious, and slight. The model does not have universal (i.e., worldwide) applicability; instead, it is limited to estimating injury severity distributions in Israel.

**Fig. 2:**
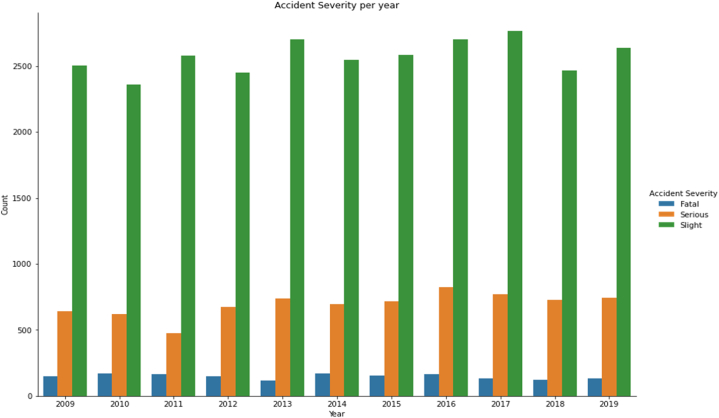
Histogram illustrating the severity of pedestrian accidents in Israel.

This is because the training process exclusively utilized data from traffic accidents in Israel during the period from 2009 to 2019. However, it is worth noting that the traffic conditions within Israel might also have relevance to other nations. Furthermore, driver and pedestrian behaviors exhibit fluctuations over time, even in a consistent environment. Accordingly, it is recommended that the model undergo continuous retraining, using new data, to uphold its accuracy and its predictive capabilities. The injury severity data presented in Fig. 2 is derived from Israeli traffic accident records between 2009 and 2019.

In order to improve road safety, it is of utmost importance to study the factors that predict severe pedestrian injuries. Multiple investigations have addressed this question, uncovering factors such as alcohol consumption, operational mistakes, attributes of the vehicles involved, collision type, environmental influences, road quality, time of occurrence, and demographic traits of the pedestrian and driver (e.g., age); see Refs. [11,12].

### 1.2 Review of the literature

Prior studies have strived to identify accident patterns through the analysis of comprehensive datasets of pedestrian fatalities. In particular, researchers have tried to uncover features with a high impact on severe injuries, while removing features with a low impact, thereby allowing the development of accurate models to predict pedestrian fatalities and severe injuries.

Al-Ghamdi [33] revealed the impact of demographic characteristics, such as age, sex, education, nationality, and social status. For example, young and old age groups of pedestrians are at an elevated likelihood of being engaged in pedestrian-vehicle crashes compared with pedestrians of intermediate age. Al-Ghamdi also identified the following risk factors: (i) pedestrian behavior prior to the accident (e.g., playing on the road, not paying attention, or failing to cross at a crosswalk); (ii) features of the environment (e.g., roads with high speed limits and residential streets); (iii) traffic control features (e.g., failed traffic light, blinking traffic light, priority sign); and (iv) vehicle type and weight. Wazana [35] also discovered that children face a greater risk of mortality and severe injury than pedestrians of other ages. Additionally, he reported a close correlation between the severity of the injury and (i) the environment (e.g., roads with high speed limits and residential streets) and (ii) the driver's demographic characteristics.

Lalika [47] formulated a Bayesian logistic regression model to identify factors that influence pedestrian fatalities and serious injuries. During the course of his investigations, he noted that pedestrians aged 65–74 were the most susceptible group in road traffic accidents. As a result, the model delineated factors that contributed to the vulnerability of older pedestrians to injury, as well as potential interconnections among these factors.

In a similar vein, Ballesteros [29] employed a logistic regression model to explore the association between the likelihood of pedestrian fatality and specific attributes encompassing the individual (age, gender), the vehicle (weight, type, engine size), and the environment (road condition, crossing characteristics). His main finding was that heavy vehicles (e.g., trucks, buses, and tractors), as well as luxury sports vehicles, were associated with a heightened risk of fatality or severe injury for pedestrians engaged in road accidents. This study also underscored the significant correlation between high-speed driving and the severity of pedestrian injuries.

The previous paragraphs provided some examples of research that aimed to pinpoint factors linked to pedestrian road-traffic injuries. A full review of this literature is beyond the scope of this paper. However, the main findings can be summarized as follows. Research has demonstrated the significance of the age and gender of the individuals involved in the collision, with clear evidence of (i) a higher incidence of high-severity injuries in both children and the elderly [2,3,24], and (ii) a greater risk of severe injury in male pedestrians than in females [6,45,46]. Other significant factors include the driver's level of intoxication through drugs or alcohol [27] and the vehicle type and weight [29,33]. In the last decade, there has been a particular emphasis on examining characteristics of the accident location, such as urban vs. interurban roads [7] and whether or not the intersection has a traffic light [25,26].

In lieu of conventional data analysis methods, machine-learning techniques have been introduced as an alternative to examine the factors contributing to injury severity [3]. This approach enables valuable insights to be extracted from voluminous and intricate datasets. Standard machine-learning methods encompass decision trees (DT) [10], Bayesian networks [11], classification and regression trees [12], and random forest [13].

In addition to using machine learning to predict the factors contributing to injury severity, the technique has a role to play in selecting suitable input data for the development of such predictive models. The predictive models can utilize highly correlated factors as input features, thereby capturing the intricate dynamics that underlie the determinants of severity. A significant challenge in crafting predictive models resides in the non-linear relationship between injury severity and the multitude of factors present in an accident—a challenge that machine-learning models can adeptly address.

Machine-learning methodologies for constructing predictive models of injury severity include DT [14], Support Vector Machine (SVM) [15], k-means clustering [16], and Artificial Neural Networks [17]. Schneider [30], for instance, formulated a predictive model of pedestrian injury severity using the logistic regression algorithm. This model integrated traffic characteristics, environmental aspects (roads and intersections), and human error. It has been argued that the logistic regression model is more helpful in building severity prediction models than other machine-learning models [31,32]. Logistic regression models have revealed a high correlation between the severity of pedestrian injury in a road traffic accident and the driver's speed and features of the accident environment [31,32].

The main machine-learning (ML) methods that have been used in crash modeling include nearest neighbor classification, decision trees, genetic programming, support vector machines, and artificial neural networks. These ML techniques have shown enhanced model performance when compared with statistical models. However, due to variations in error measurement methods across studies, a direct comparison of the performance of these different techniques is not feasible. The key explanatory variables used in modeling accident severity, in decreasing order of importance, are as follows: road-environmental factors, human factors, collision characteristics, and vehicle-related factors. In crash-based models, the most powerful explanatory variables are gender, age, vehicle type, vehicle age, season, time of day, and day of the week [52,53].

Among the analytical machine-learning algorithms, K-Nearest Neighbor (KNN) is a non-parametric statistical approach for classification and regression [52,55]. It predicts by identifying k neighbors in proximity to a response variable value based on their calculated distance. The anticipated response variable value is then determined from the k neighbors. Although KNN does not make assumptions about the functional form of the data [56], it can be sensitive to local data structure. Decision Trees (DTs) construct classification or regression models in the form of a tree that predicts the response variable's value by selecting the explanatory variable with the “best” split at each level. While they are intuitive, the challenge with DTs lies in overfitting, which compromises generalization [54]. The Random Forest (RF), designed to mitigate the overfitting issue encountered with DTs, is a collection of uncorrelated decision trees that collaborate to make predictions [57]. Since the trees are assembled using a bootstrap aggregation method, they remain uncorrelated. The aggregate prediction from an RF outperforms individual trees in terms of accuracy. While not as straightforward to interpret as a DT, an RF offers a simple way of assessing the importance of each explanatory variable [58].

It is important to note that this research conducts its own feature selection process instead of relying on features identified in previous literature. The chosen algorithm for predicting injury severity is dependent on the input data, which in turn are derived by applying feature-selection algorithms to Israeli road-traffic accident data. The motivation behind developing a new injury-severity prediction algorithm was to explore a wide array of machine-learning approaches, some of which had not been utilized in prior research. Specifically, while previous models for predicting injury severity were generally rooted in various regression-based machine-learning algorithms, the findings of the current study suggest that decision-tree algorithms are likely to be better suited to this type of input data. A further contribution of this study is that it introduces a tool to calculate the potential financial savings resulting from policy changes, demonstrating a practical application of the model. To the best of our knowledge, previous studies have not analyzed the financial savings due to implementing policies to reduce severe pedestrian injuries. To sum up, the primary objectives of this study are to identify a set of features predicting injury severity among pedestrians in Israeli traffic accidents and to utilize these features to construct a machine-learning model that can offer practical solutions.

### 1.3 Objectives of the research

The objectives of this study are threefold.

1. To pinpoint features that have a strong association of considering how seriously pedestrians are hurt in traffic collisions on roads.
2. To formulate a highly accurate predictive model for determining the severity of pedestrian injuries.

## 2 An introduction to the research methodology

**Fig. 3:**
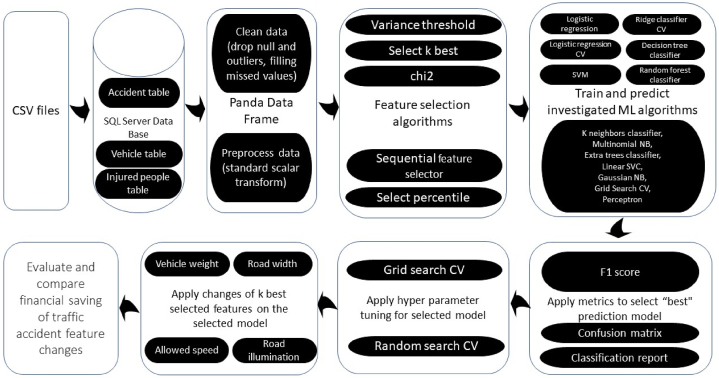
The study flowchart.

This study uses similar methodology to that applied in our previous study on machine-learning models for reducing the severity of bicyclist road traffic injuries [61]. The starting point of the present study was to produce a list of potential characteristics that might be associated with pedestrian injury severity. The next stage entailed selecting the most predictive features from this list and using them as input data when training a set of candidate predictive machine-learning algorithms. The flowchart in Fig. 3 depicts the sequence of actions for crafting and selecting the optimal predictive machine-learning model [61].

## 3 Data

The input data, sourced from Israel's Central Bureau of Statistics, comprised 47,432 traffic crashes involving pedestrians between 2009 and 2019. Of this total, 1392 crashes were fatal, and 46,040 involved a non-fatal injury to the pedestrian. The dataset contained 56 variables, including the unique crash ID, the year, date, and time of the crash, pedestrian and driver attributes (e.g., age group), accident location, and road features.

The Israeli control authorities maintain Israeli traffic accident data using 14 different CSV files, each with a distinctive structure. In order to streamline, enhance, and improve the data querying process, and to ensure the integrity of incoming data, the set of files was loaded into an SQL Server relational database utilizing the data transformation utilities provided by SQL Server. Within this framework, three central entities are established: Accident, Injured Person, and Vehicle. Both the Injured Person and Vehicle tables were furnished with Accident ID columns, aligning with those in the Accident table. This interconnected design enabled data synchronization across the tables.

**Fig. 4:**
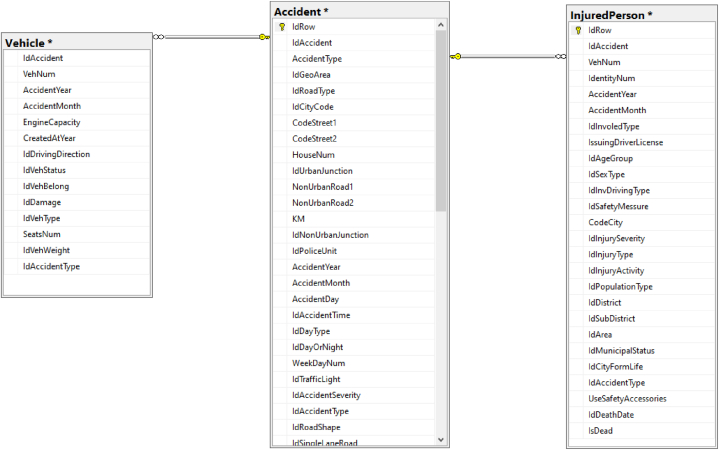
Variables within each domain table in the database.

To ensure data integrity, stringent data integration protocols were enforced. These included appropriate column data type assignments (such as integers, floats, and dates), establishment of foreign and unique indexes and default values. Foreign key indexes were also applied to the Vehicle and Injured Person tables. For a visual representation of the variables encapsulated within each domain table, please consult Fig. 4. Note that this research uses the same database structure as that employed in our previous study [61], but in the present study, we extracted data that were related to the pedestrian as the injured party.

The Accident table included a variety of attributes, the classification of the road type and the geographical accident coordinates (longitude and latitude). The Vehicle table captured details pertaining to the vehicle(s) implicated in the road traffic crash, for example, vehicle type and weight. The Injured Person table documented information related to the individuals involved in the accident, including both drivers and pedestrians. This category includes an injury range, from not injured to fatal, serious, and slight, gender, and age group.

## 4 Preparation of data and process of selecting features

The predictive models (described in Section 6) were constructed using the Jupyter Notebook platform, which operates within a web-based environment and employs the Python 3.9 programming language for calculations. Jupyter Notebook serves as a valuable framework for machine learning. It offers functionality with respect to data analysis, model development, evaluation, and visualization, specifically tailored to research in machine learning. Moreover, it provides a wide array of integrated features optimized for machine learning purposes. The data extracted from the logical view of the SQL Server were imported into a Python Pandas Data Frame for preprocessing and assessment. Specifically, the following actions were taken.

1. Eliminate records where the severity of pedestrian injury is unidentified.
2. Substitute empty and NULL values of features with a suitable average value (either mean or median) for the respective column. Mean imputation is used when the missing values in a dataset column are numerical and the variable's distribution is roughly normal. When the distribution is skewed, median imputation is preferable because it is less susceptible to outliers. This study applied the Imputer algorithm from the sklearn preprocessing library to fill in the missing values.
3. Detect and remove outliers. The interquartile range (IQR) methodology and a visual examination of the data were used to identify outliers. IQR is a statistic that expresses the difference between a dataset's first and third quartiles, IQR = Q75 - Q25. Various feature-selection algorithms were employed to identify the most influential attributes, which would serve as inputs to the machine-learning algorithms. The following four feature-selection algorithms (where the names are those used in the sklearn feature selection module) were executed, with the outcome of each method (i.e., the list of influential features) provided underneath:

### 4.1 VarianceThreshold

The Variance Threshold method was implemented by retaining features with a variance in excess of 0.05, as low-variance features do not contribute significantly to modeling. It is recommended to use a small threshold value, close to zero [50]. Applying this criterion, the 35 features outlined in Table 1 were chosen, meaning that these features exhibit a strong association with the severity of pedestrian injuries.

**Table 1:** Attributes displaying the most robust correlation with injury severity, as determined by the VarianceThreshold algorithm.

#	Explanatory variable	Values
1	Gender of the pedestrian	Male, female
2	Gender of the driver	Male, female
3	The pedestrian's religion	Jewish, non-Jewish, not specified, other
4	The driver's religion	Jewish, non-Jewish, not specified, other
5	Location of the pedestrian's crossing	On crosswalk with traffic light, on crosswalk without traffic light, not on crosswalk and far from an intersection, on crosswalk next to intersection, not specified
6	Engine capacity	Less than 125 cc, between 125 and 500 cc, from 501 cc to 800 cc, more than 800 cc
7	Severity of damage to the vehicle	Minor, medium, severe, no damage
8	Vehicle type	Freight (>34.0 tons total weight), motorcycle up to 50 cc, motorcycle 51–250 cc, motorcycle 251–500 cc, motorcycle >500 cc, minibus, bus, cab, work vehicle, tractor, bicycle, train
9	Vehicle weight	Less than 2.0 tons, 2.0–2.9, 3.0–3.5, 3.6–4.0, 4.1–5.9, 6.0–7.9, 8.0–9.9, 10.0–12.0, 12.1–12.9, 13.0–15.9, 16.0–19.0, 19.1–25.9, 26.0–30.0, 30.1–32.0, 32.1–33.9, 34.0–40.0, 40.1–56.0, >56.0
10	Weekday	Sunday, Monday, Tuesday, Wednesday, Thursday, Friday, Saturday
11	Traffic control	No control, working traffic light, failed traffic light, blinking yellow, stop sign, priority sign, not specified
12	Road width	Up to 5 *m*, 5–7 *m*, 7–10 *m*, 10–14 *m*, over 14 *m*
13	Weather conditions	Clear, rainy, hot, foggy, not specified
14	Maximum allowed speed	50 km/h, 60 km/h, 70 km/h, 80 km/h, 90 km/h, 100 km/h
15	Section of road	Entrance to an interchange, exit from an interchange, steep slope, sharp curve, on a tunnel bridge/railroad junction, straight road, junction, bus stop, public transport route, other
16	Location of the accident	Urban at an intersection, urban not at an intersection, non-urban at an intersection, not urban not at a crossroads
17	District	Jerusalem, the North, Haifa, the Center, Tel Aviv, the South, Yehuda mountains, Gaza Strip
18	Cause of the accident	Driver behavior, passenger behavior, pedestrian behavior, motorcyclist behavior, cyclist behavior, vehicle malfunctioning
19	Direction of car (i.e., whether it is coming from the pedestrian's left or right) when the pedestrian crosses the road	From right to left, from left to right, unknown
20	Road signpost	Defective/missing marking, defective/missing signpost, signpost is required and it is not missing or defective, unknown
21	Road surface conditions	Dry, wet from water, wet from slippery material, covered with mud, covered with sand, not specified
22	Type of road	One-way road, two-way road with separation, two-way road without separation, not specified
23	Type of carriageway division	With separation fence, without separation fence, not specified
24	Nature of the pedestrian's crossing	Sudden, from a hidden place, normal, not specified
25	Illumination on the road	Daylight, night without illumination, night with illumination
26	Day/night	Day, night
27	Age of the pedestrian	0-18, 19–24, 25–34, 35–44, 44–54, 55–64, more than 64
28	Condition of road shoulders	Good condition, bad condition, rough road
29	Driver's experience (years)	Up to 2, 2–5, 6–10, 11–20, more than 20
30	Safety measures in the vehicle	Seatbelts, helmets, child seat, no safety measures used
31	Time of day	Morning peak, off-peak, afternoon peak, evening, night
32	Season	Spring, summer, autumn, winter
33	Number of lanes on the road (in any direction)	1, 2, 4, 6, more than 6
34	Road integrity	Broken road, bad road margins, no road margins and broken road, road without defects, unknown
35	Type of day	Normal, pre-festive, festive

### 4.2 SelectKBest

The SelectKBest algorithm scores the attributes and retains only the top k highest-scoring features while discarding the rest [50]. Employing this algorithm resulted in the identification of the subsequent array of features: pedestrian crossing location, engine capacity, vehicle type, vehicle weight, road illumination, weather conditions, road surface state, maximum permissible speed, road section, traffic control, accident location, vehicle safety measures, driver's experience (in years), day of the week, and pedestrian age.

### 4.3 SelectPercentile

The SelectPercentile algorithm functions by choosing features based on a percentile of the top scores. This algorithm identified the following attributes: pedestrian's time of day, pedestrian's age and gender, pedestrian's crossing location, road's lane count, road width, road illumination, weather conditions, road surface state, maximum permissible speed, road type, traffic control, district, vehicle safety measures, driver's experience in years, and the day of the week.

### 4.4 SequentialFeatureSelector

The sequential feature selector operates by incrementally adding (forward selection) or excluding (backward selection) features to construct a subset of features in a stepwise manner. During each iteration, this estimator selects the most optimal feature for inclusion or removal, based on the cross-validation score of a given estimator. In the context of unsupervised learning, the sequential feature selector [50]. The attributes chosen by this algorithm were: maximum permissible speed, vehicle type, pedestrian's crossing location, road illumination, vehicle safety measures, road's lane count, accident cause, accident location, day type, pedestrian's age and gender, traffic control, district of accident occurrence, and vehicle type.

To consolidate the outcomes of the four aforementioned algorithms, we opted for the 20 features that occurred with the highest frequency across the algorithms. The conclusive list encompassed (see Table 2): vehicle type, vehicle weight, pedestrian's crossing location, road surface state, direction of the approaching car in relation to the pedestrian's movement, accident location, road illumination, lane count (in any direction), day type, time of day, pedestrian's age group, pedestrian's gender, road width, driver's experience, traffic control, accident cause, maximum permissible speed.

**Table 2:** Final set of attributes utilized as input data for the machine-learning algorithms.

#	Explanatory variable	Values
1	Vehicle type	Freight (>34.0 tons total weight), motorcycle up to 50 cc, motorcycle 51–250 cc, motorcycle 251–500 cc, motorcycle >500 cc, minibus, bus, cab, work vehicle, tractor, bicycle, train
2	Vehicle weight	Less than 1.9 tons, 2.0–2.9, 3.0–3.5, 3.6–4.0, 4.1–5.9, 6.0–7.9, 8.0–9.9, 10.0–12.0, 12.1–12.9, 13.0–15.9, 16.0–19.0, 19.1–25.9, 26.0–30.0, 30.1–32.0, 32.1–33.9, 34.0–40.0, 40.1–56.0, >56.0
3	Location of the pedestrian's crossing	On crosswalk with traffic light, on crosswalk without traffic light, outside crosswalk far from an intersection, crosswalk next to intersection, not specified
4	Road surface conditions	Dry, wet from water, wet from slippery material, covered with mud/covered with sand, not specified
5	Direction of the car that is coming from the pedestrian's left or right when the pedestrian crosses the road	From right to left, from left to right, unknown
6	Location of the accident	Urban at a junction, urban not at an intersection, non-urban at an intersection, not urban not at a crossroads
7	Illumination on the road	Daylight, night without illumination, night with illumination
8	Number of lanes on the road in any directions	1, 2, 4, 6, more than 6
9	Type of day	Normal, pre-festive, festive
10	Time of day	Morning peak, off-peak, afternoon peak, evening, night
11	Month of the year	1–12
12	Age group of pedestrian	Less than 14–15, 19–24, 25–34, 35–44, 44–54, 55–64, more than 64
13	Age group of driver	Less than 14–15, 19–24, 25–34, 35–44, 44–54, 55–64, more than 64
14	Gender of the pedestrian	Male, female
15	Gender of the driver	Male, female
16	Width of the road	Up to 5 *m*, 5–7 *m*, 7–10 *m*, 10–14 *m*, over 14 *m*
17	Driver's experience (years)	Up to 2, 2–5, 6–10, 11–20, more than 20
18	Type of traffic control	No control, working traffic light, failed traffic light, blinking yellow, stop sign, priority sign, not specified
19	Cause of the accident	Driver behavior, passenger behavior, pedestrian behavior, motorcyclist behavior, cyclist behavior, vehicle malfunctioning
20	Permitted maximum speed	50 km/h, 60 km/h, 70 km/h, 80 km/h, 90 km/h, 100 km/h

The five variables that have the highest impact on the severity of pedestrian injury, as determined by assessing the average level of impact across all four feature-selected algorithms, were as follows: vehicle type, vehicle weight, location of the pedestrian's crossing, site of the accident, and maximum allowed speed. The second level of variables (i.e., variables that have less impact on the severity of pedestrian injury) includes the pedestrian's age group, traffic control, and the driver's experience (years).

## 5 The relationship between features and injury severity

This section provides graphical representations of the relationship between selected features (those that strongly correlate with injury severity) and the level of injury severity. The feature selection algorithms did not take into account the co-correlation between variables when selecting the variables. The other variables (i.e., those not depicted in the following graphs) showed a weaker correlation with injury severity. The inference drawn from both these graphs indicates that there is a greater likelihood of severe pedestrian injuries with heavier vehicles. These findings receive support from various recent studies [29,32,33,36,37].

**Fig. 5:**
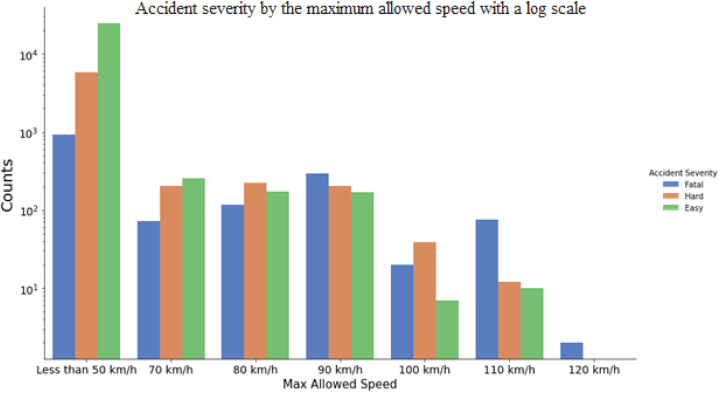
Accident severity by the maximum allowed speed, where the accident counts are plotted on a log scale.

Fig. 5 illustrates the injury severity distribution as a function of the maximum allowed speed, demonstrating that the proportion of fatal accidents is higher when the speed limit exceeds 70 km per hour. This result accords with previous research [40,41].

**Fig. 6:**
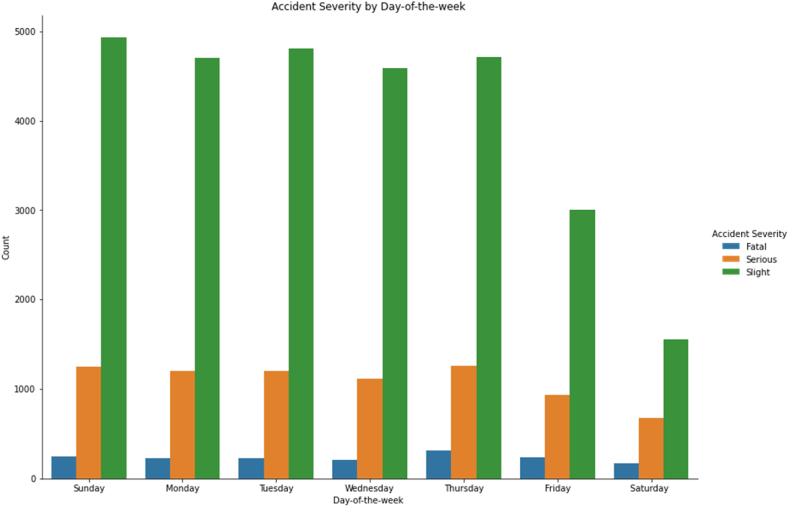
Accident severity by day of the week.

Fig. 6 presents the distribution of pedestrian injury severity by day of the week. It shows that more road accidents involving pedestrians occur on weekdays than at weekends (Sunday is a workday in Israel). This finding may be due to the tendency of religious citizens in Israel to avoid travel on the Sabbath (Saturday) and the eve of the Sabbath, resulting in less heavy traffic on these days. This result contrasts with Europe and the USA, where road accidents involving pedestrians are higher on weekends [43,44].

**Fig. 7:**
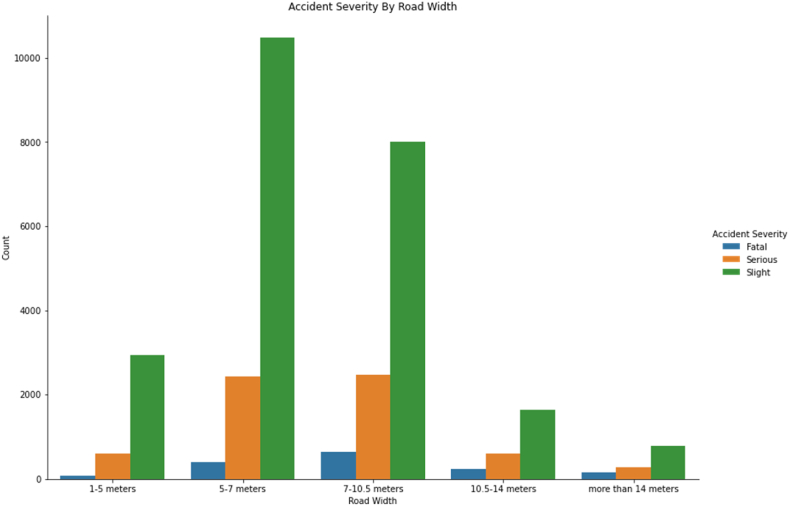
Accident severity by road width.

Fig. 7 examines the severity of injuries in relation to road width. It can be seen that the proportion of fatal and moderate injuries increases with road width. For example, a 7–10.5 m-wide road has a higher proportion of fatalities than narrower roads. These results resemble findings in the Western world generally [44].

**Fig. 8:**
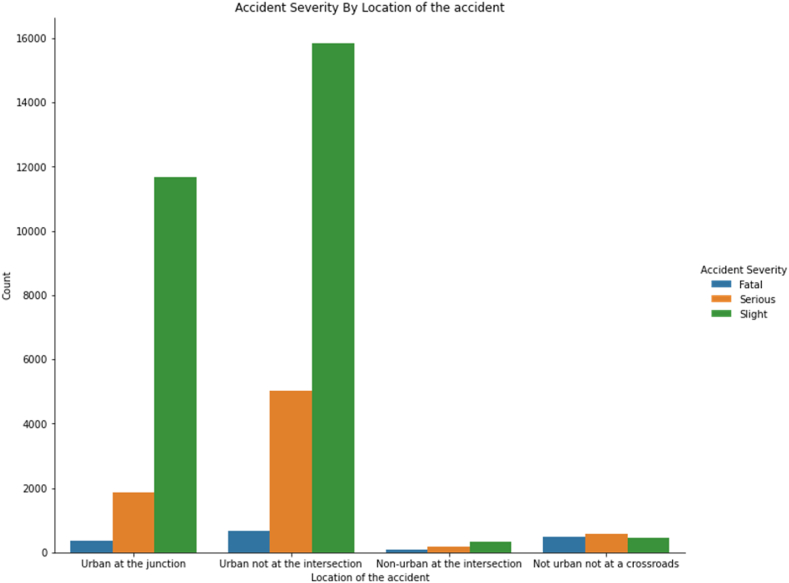
Accident severity by location of the accident.

Fig. 8 presents the distribution of injury severity as a function of the accident's location. It reveals that the proportion of fatalities is higher when the accident is not associated with a pedestrian's attempt to cross the road. This discovery aligns with previous findings that the chances of pedestrian survival increase significantly when an accident occurs in a city at a junction with an orderly crosswalk [31,37].

**Fig. 9:**
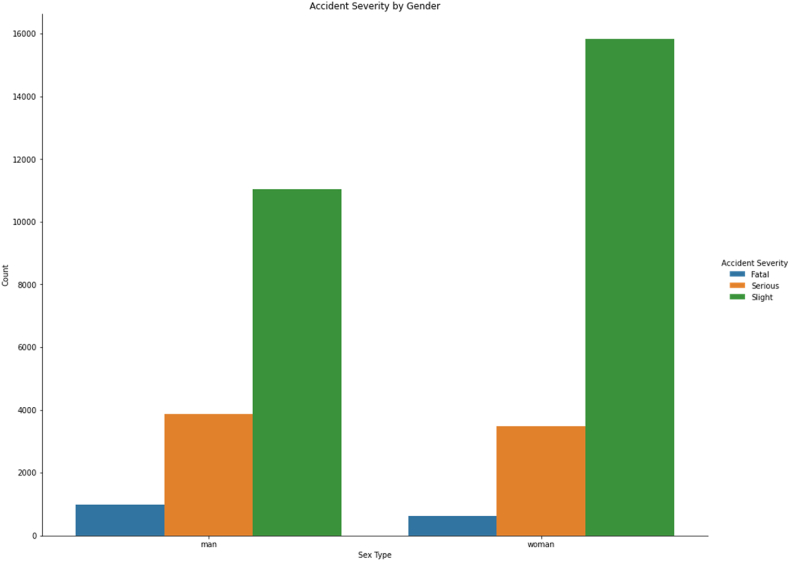
Accident severity by the gender of the pedestrian.

Fig. 9 shows the distribution of the severity of injuries in relation to the gender of the pedestrian. It reveals that women sustain more injuries than men, but that the rate of severe injury is lower in women (see Refs. [45,46]).

## 6 Assessment and appraisal of machine-learning models

This section investigates a range of machine-learning classification models to ascertain the most predictive model for evaluating injury severity. This approach is commonly employed in machine-learning research, thus allowing the selected machine-learning algorithm to be labeled as a “cutting-edge” model despite its limited prior use. The evaluated models comprised the following: Logistic Regression, Logistic Regression CV, Support Vector Machine (SVM), Linear Support Vector Classifier (SVC), Naive Bayes, Gaussian Naive Bayes, Ridge Classifier, Ridge Classifier CV, Decision Tree Classifier, Random Forest Classifier, Extra Tree Classifier, Perceptron, and K-nearest Neighbors. The choice of this particular set of algorithms was influenced by our past experience with their application. However, it is important to acknowledge that limiting the investigation to this selection could constitute a shortcoming of our study. The effectiveness of each model was assessed using the classification report, which contained the computed values of the following metrics: accuracy, precision, recall, and F1 score. The model displaying the highest values across these four metrics was identified as the “best” predictive model under investigation. Performance metrics such as accuracy, precision, recall, and F1 score are frequently utilized to gauge the efficacy of classification models. These metrics can be calculated using a confusion matrix, a table that contrasts predicted labels with actual labels to highlight a model's performance. The confusion matrix was employed to extract the aforementioned performance measures, as described in the subsequent paragraphs.

Accuracy reflects the number of data instances within a particular class that are accurately assigned the correct label, relative to the total number of data instances of that class. The overall accuracy is then computed as the average accuracy across all classes [51], which is three in the case of the present study. Thus, accuracy was computed using the following formula:

Accuracy = (True Positives_fatal + True Positives_serious + True Positives_slight + True Negatives_fatal + True Negatives_serious + True Negatives_slight)/(True Positives_fatal + True Positives_serious + True Positives_slight + True Negatives_fatal + True Negatives_serious + True Negatives_slight + False Positives_fatal + False Positives_serious + False Positives_slight + False Negatives_fatal + False Negatives_serious + False Negatives_slight) [61].

Precision is the proportion of correctly predicted instances of a given class out of all the instances predicted to have that class, averaged across all classes [51]. The general formula for precision is True Positives/(True Positives + False Positives). For our concrete case, we calculate precision according to:

Precision = (True Positives_fatal + True Positives_serious + True Positives_slight)/((True Positives_fatal + True Positives_serious + True Positives_slight) + (False Positives_fatal + False Positives_serious + False Positives_slight)) [61].

Recall is the number of correctly predicted instances of a given class out of the number of actual instances of that class [51]. The general recall formula is True Positives/(True Positive + False Negatives). For our concrete case, we calculate precision as:

Recall = (True Positives_fatal + True Positives_serious + True Positives_slight)/((True Positives_fatal + True Positives_serious + True Positives_slight) + (False Negatives_fatal + False Negatives_serious + False Negatives_slight)) [61].

The F1 score is a balanced combination of precision and recall, calculated as a harmonic mean [51]. The F1 score is computed as follows:

F1 = (2 * Precision * Recall)/(Precision + Recall) [61].

The final metric that is reported in a classification report is the “support”, which refers to the count of actual occurrences of a specific class within the provided dataset. When the support is imbalanced in the training dataset, it could denote potential flaws in the classifier's reported scores and may signal the need for rebalancing or sampling. The support score remains consistent across models, and therefore it is not useful as a means of selecting the “best” model; however, it serves as a diagnostic tool for the evaluation process.

The machine-learning models were developed using the scikit-learn package, which allows calculation of the aforementioned performance metrics. The initial step involved partitioning the clean, raw data into 2 different sets: training and testing. The training dataset encompassed 80 % of the entire dataset, with the remaining 20 % designated for testing. All data manipulations, such as shuffling and transformations, were exclusively applied to the training dataset. The test data remained untouched and were employed during the model evaluation phase to generate the classification reports. Table 3 outlines the performance metrics that were attained by the investigated models.

**Table 3:** Accuracy, precision, recall, and F1 score obtained from the test data.

Classification algorithm name	Accuracy	Precision	Recall	F1 score
Logistic Regression	0.96	0.96	0.90	0.92
Logistic Regression CV	0.97	0.96	0.90	0.93
SVM	0.97	0.96	0.91	0.94
Linear SVC	0.94	0.90	0.75	0.79
Naive Bayes	0.89	0.69	0.73	0.71
Gaussian Naive Bayes	0.90	0.74	0.80	0.75
Ridge Classifier	0.93	0.81	0.66	0.67
Ridge Classifier CV	0.93	0.82	0.66	0.67
Decision Tree Classifier	0.96	0.93	0.95	0.94
Random Forest Classifier	0.98	0.99	0.95	0.97
Extra Tree Classifier	0.98	0.99	0.96	0.98
Perceptron Algorithm	0.93	0.82	0.84	0.83
K-nearest Neighbors	0.89	0.81	0.74	0.77

Based on the performance metrics in Table 3, Extra Tree Classifier algorithm was identified by us as the most predictive approach for forecasting the severity of pedestrian injuries due to road traffic accidents occur in Israel. To enhance the accuracy of the Extra Tree Classifier, we employed the GridSearchCV algorithm from the scikit-learn package [50] to determine optimal hyperparameter values. Firstly, GridSearchCV evaluated the optimum amount of trees required for the Extra Tree Classifier across a range spanning from 10 to 5000. The conclusion was that the optimal count was 500. Following this, GridSearchCV investigated the impact of the number of samples at decision tree junctions before introducing a split, referred to as “min_samples_split.” Values of min_samples_split ranging from 2 to 15 were tested, resulting in an optimal value (i.e., yielding the highest accuracy) of 10. As a result of these adjustments to the Extra Tree Classifier, its accuracy was elevated to 0.989 (relative to a previous value of 0.985).

This study employed a machine-learning approach. An alternative methodology for anticipating the severity of pedestrian road-traffic injuries is to use statistical methods to identify traffic patterns across various scales: daily patterns, patterns on different days of the week, seasonal variations, and so on. These scales are generally simpler, quicker, and more cost-effective to implement than machine-learning methods. However, their accuracy is somewhat limited as they cannot handle extensive multivariate data as effectively. Convolutional neural networks have established themselves as reliable leaders in image recognition and analysis. A common application of these networks in the field of transportation is congestion detection, which utilizes images captured by road surveillance cameras. Recurrent neural networks, on the other hand, are designed to handle time-series data or observations collected over specific time intervals. Traffic patterns serve as a fitting illustration of such observations [60].

## 7 Discussion

### 7.1 Contributing to the research

The main goal of this research was to discover and establish the most accurate machine-learning model for predicting the severity of injuries suffered by pedestrians involved in road accidents. According to this study, machine-learning models play a crucial role in managing complex and substantial datasets. These algorithms offer an automated means of uncovering patterns and relationships within data, a task that can be challenging or infeasible when attempted manually.

The Extra Tree Classifier method demonstrated exceptional performance, with an accuracy of 0.98, precision of 0.99, recall of 0.96, and F1 score of 0.98. Another promising option for assessing the severity of pedestrian road-traffic injuries is the Random Forest Classifier algorithm, which closely trailed the Extra Tree Classifier in terms of its performance (accuracy of 0.98, precision of 0.99, recall of 0.95, and F1 score of 0.97).

This study identified the following features as having the greatest value as input data to predict pedestrian injury severity: vehicle type, vehicle weight, pedestrian's crossing location, road surface conditions, direction of approaching car in relation to pedestrian's movement when crossing the road, accident location, road illumination, lane count in any direction, day type, time of day, month, pedestrian's age group, driver's age group, pedestrian's gender, driver's gender, road width, driver's experience in years, type of traffic control, accident cause, and maximum allowable speed. Broadly speaking, these findings validate the main conclusions of previous literature examining the factors that influence injury severity [29,32,33,36,37,41,[43], [44], [45], [46],^49^]. However, the present study did not uncover any notable influence of driver alcohol consumption – a parameter that is well-recognized in the United States and European nations, and has been established as a leading contributor to pedestrian fatalities [4].

In prior studies of a similar nature, researchers have favored algorithms such as logistic regression [29,31,32], K-Nearest Neighbor [55], Decision Trees [56], and Random Forest [57]. The Extra Tree Classifier is a decision tree technique that significantly enhances the fundamental design of conventional decision trees. It adds a small amount of randomness to decision tree building, which can reduce overfitting and enhance generalization performance. To be more precise, it uses random selection rather than a fixed set of criteria to divide the data into subsets of features and thresholds at each node. With only a small number of hyperparameters to adjust, the Extra Tree Classifier is computationally efficient and can be trained on huge datasets. This makes it suitable for tasks requiring little processing power and for high-dimensional datasets. Relative to conventional decision trees, the Extra Tree Classifier is more resistant to noise and outliers in the data. This is because the random selection procedure makes it is less likely to overfit these occurrences. The Extra Tree Classifier is readily parallelizable, meaning that it may be used with distributed computing clusters or multiple processors, thereby resulting in a shorter training period. Finally, the Extra Tree Classifier provides a measure of feature significance, meaning that it can identify the most useful features and can enhance model interpretability.

The benefits of employing tree-based learning algorithms include their capacity to train models on extensive datasets and their ability to accommodate both quantitative and qualitative input variables. Additionally, tree-based models are better able to deal with redundant variables and variables with high correlation, which may lead to overfitting in other learning algorithms. Trees also entail very few parameters that require tuning when training the model, and they perform relatively well in the presence of outliers or in cases where the dataset contains missing values.

### 7.2 Limitations and future research

This study has limitations due to the database structureThe study was applied only to a single geographical location (Israel), due to the need to access to the relevant data, meaning that some of the conclusions may not be applicable to other countries. However, due to its method of operation, we anticipate that our conclusions about the suitability of the Extra Tree Classifier are likely to hold for models developed using data obtained in other countries.

In addition to the aforementioned geographical limitation, the findings of this study may be limited to the time period encompassed by the database (2009–2019). Therefore, the machine-learning model should be retrained on a regular basis using the latest traffic accident data.

This study did not examine the potential of a range of ensemble classifiers, including the Voting Classifier, Stacking Classifier, AdaBoost, Gradient Boosting Classifier, and Histogram-based Gradient Boosting Classifier. Ensemble methods strive to amalgamate predictions from multiple base estimators constructed through a specific learning algorithm. This amalgamation aims to enhance generalizability and resilience compared to a solitary estimator. Additionally, the research did not delve into the efficacy of classification algorithms within the realm of deep learning, nor did it examine image processing techniques. These subjects warrant exploration in forthcoming studies.

### 7.3 Conclusion

In order to formulate strategies to enhance road user safety, it is essential to develop the ability to predict the circumstances under which severe injuries are most likely to be sustained. This investigation underscored specific factors that exert significant influence over the severity of pedestrian injuries resulting from traffic incidents in Israel based on data collected between 2009 and 2019. The most influential features were: vehicle type, vehicle weight, the location at which the pedestrian crosses the road, the geographical coordinates of the accident, and the maximum permissible speed. The Extra Tree Classifier demonstrated the highest level of efficacy in predicting the seriousness of injuries sustained by pedestrians involved in traffic accidents in Israel.

## Data availability statement

The data associated with this study is public available for researcher. In order to access this data you should fill this form https://survey.gov.il/he/License_to_use_PUF_CBS.

## Additional information

No additional information is available for this paper.

## CRediT authorship contribution statement

**Amir Elalouf:** Validation, Supervision, Project administration, Conceptualization. **Slava Birfir:** Writing – review & editing, Writing – original draft, Visualization, Software, Investigation, Formal analysis, Data curation. **Tova Rosenbloom:** Supervision, Methodology, Data curation, Conceptualization.

## Declaration of competing interest

The authors declare that they have no known competing financial interests or personal relationships that could have appeared to influence the work reported in this paper.
